# Serum IgG level is a predicting factor for the response to neoadjuvant chemotherapy in patients with esophageal squamous cell carcinoma

**DOI:** 10.1186/s12957-021-02290-7

**Published:** 2021-07-19

**Authors:** Seiichi Nakaya, Ryo Ogawa, Shunsuke Hayakawa, Shiro Fujihata, Tomotaka Okubo, Hiroyuki Sagawa, Tatsuya Tanaka, Hiroki Takahashi, Yoichi Matsuo, Shuji Takiguchi

**Affiliations:** Department of Gastroenterological Surgery, Nagoya City University Graduate School of Medical Sciences, 1-Kawasumi, Mizuho-cho, Mizuho-ku, Nagoya City, Aichi 467-8602 Japan

**Keywords:** Immunoglobulin G, Esophageal squamous cell carcinoma, Neoadjuvant therapy, Chemotherapy

## Abstract

**Background:**

Despite the established oncological benefits of neoadjuvant chemotherapy for esophageal squamous cell cancer, not all cases demonstrate benefit. Hence, predicting the response to chemotherapy before treatment is desirable. Some reports have shown that immune factors are related to the chemotherapy response. This study aimed to investigate the utility of serum IgG levels for predicting chemotherapy response.

**Methods:**

Among the patients who underwent esophagectomy after neoadjuvant chemotherapy at Nagoya City University Hospital between December 2012 and June 2019, 130 cases were included in this study. Response to chemotherapy and pretreatment serum IgG levels were examined in 77 cases. FP (5-fluorouracil and cisplatin) therapy or DCF (docetaxel, cisplatin, and 5-FU) therapy was performed as neoadjuvant chemotherapy. DCF therapy was selected for patients aged <75 years, who could be safely administered chemotherapy based on their medical history.

**Results:**

This study divided cases into two groups: the effective response group (PR) and ineffective response group (SD and PD). We classified 1, 37, and 39 cases as PD, PR, and SD, respectively. None of the cases were classified as CR. The effective response group had significantly lower serum IgG levels than the ineffective response group (*p* < 0.001). The cutoff serum IgG value was determined to be 1087 mg/dL. The low IgG group had significantly more cases who had effective response to chemotherapy compared with the high IgG group (odds ratio [OR] = 9.009; 95% confidence interval [CI] = 2.974–30.157; *p* < 0.001). Univariate and multivariate analyses revealed serum IgG level to be an independent predictor for response to chemotherapy (*p* = 0.001). Furthermore, cases with effective pathological response had significantly lower pretreatment serum IgG levels than those who did not (*p* = 0.006).

**Conclusions:**

Our finding showed that serum IgG levels can be an independent predictor of the response to neoadjuvant chemotherapy for esophageal squamous cell carcinoma.

**Trial registration:**

This retrospective study was approved by the review board of Nagoya City University Graduate School of Medical Sciences (reception number: 60-18-0008).

## Introduction

Esophageal cancer, which ranks seventh in terms of incidence and sixth in terms of overall mortality worldwide, has remained one of the most common malignancies, with esophageal squamous cell cancer (ESCC) being the dominant histological type in East Asia [1].

The oncological benefits of neoadjuvant chemotherapy and chemoradiotherapy for ESCC have had a major impact on clinical practices [2–4]. Japanese guidelines for the treatment of ESCC have shown that neoadjuvant chemotherapy comprising 5-fluorouracil and cisplatin (FP), established by the JCOG9907 trial, was beneficial for patients with clinical stage II/III ESCC [5]. Furthermore, a randomized controlled phase III trial comparing FP; docetaxel, cisplatin, and 5-fluorouracil (DCF); and FP–radiotherapy as neoadjuvant treatment for clinical stage II or III esophageal cancer (JCOG1109 trial) is currently being conducted, with our department also conducting DCF therapy [6].

Studies have reported that FP and DCF therapy for ESCC had response rates of 38% and 70%, respectively [5, 7]. In some cases, however, neoadjuvant chemotherapy may not be effective. As such, predicting responses to chemotherapy before treatment is desirable.

Currently, immune checkpoint inhibitors, such as PD-1 inhibitors, have attracted attention for cancer treatment [8, 9]. Accordingly, immune factors have been found to be important in cancer prognosis and response to chemotherapy, with reports showing a relationship between lymphocyte infiltration into the tumor and chemotherapy response, as well as findings suggesting that immunological factors, such as the neutrophil–lymphocyte ratio (NLR), are effective in predicting responses to chemotherapy [10–15]. Indeed, predicting responses to chemotherapy through general tests, such as blood tests, as well as the identification of several markers, will be of considerable benefit. Some reports have shown that IgG, one of the immunolocalize factors, is involved in cancer immunity through antibody-dependent cellular cytotoxicity (ADCC) [16]. Therefore, the current study aimed to investigate the utility of serum IgG levels in predicting the response to chemotherapy.

## Materials and methods

### Patients

**Fig. 1:**
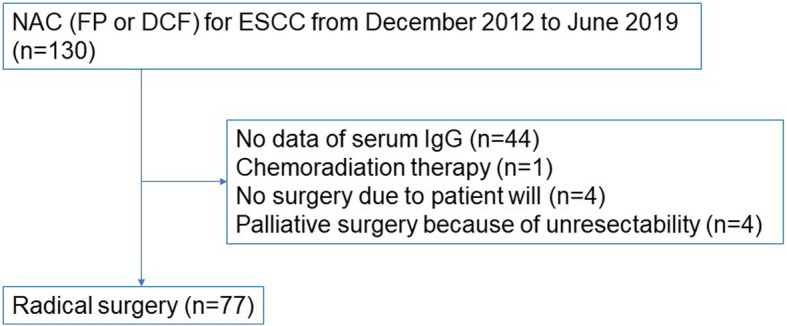
Flow chart for study inclusion

Among the patients who underwent esophagectomy after neoadjuvant chemotherapy at Nagoya City University Hospital between December 2012 and June 2019, 130 cases who (1) had histologically confirmed ESCC following biopsy and (2) received neoadjuvant chemotherapy were included in this study. Those who received chemotherapy due to unresectable ESCC (e.g., clinical M1 disease) were excluded. Among the 130 cases, 44 had no serum IgG measurements prior to neoadjuvant chemotherapy, while 1 underwent chemoradiation therapy, leaving 85 cases. Moreover, four cases who did not undergo surgery at the patient’s request and another four who underwent palliative surgery due to intraoperative unresectable findings were excluded. Ultimately, 77 cases were enrolled herein (Fig. 1). The Union for International Cancer Control Tumor-Node-Metastasis Classification (7th edition) was used for staging.

This retrospective study was approved by the Institutional Review Board and the Ethics Committee of Nagoya City University Graduate School of Medical Sciences (reception number 60-18-0008), and informed consent for publication was obtained from all patients prior to the therapy.

### Neoadjuvant chemotherapy

By 2016, our institution had introduced DCF as neoadjuvant chemotherapy for ESCC. DCF therapy was selected for patients below 75 years old in whom chemotherapy could be safely administered based on their medical history. For FP therapy, cisplatin (80 mg/m^2^) was administered on day 1, while 5-FU (800 mg/m^2^) was administered on days 1–5, with one course lasting 28 days. For DCF consisting of FP and docetaxel, cisplatin (70 mg/m^2^) and docetaxel (70 mg/m^2^) were administered on day 1, while 5-FU (700 mg/m^2^) was administered on days 1–5, with one course lasting 28 days [14]. A total of two chemotherapy courses had been planned, while surgery was performed approximately 4–6 weeks after chemotherapy. When grade 3 or above adverse events were observed, the dose was reduced by up to 25%. However, when serious myelosuppression, renal dysfunction, or impaired liver function were observed, chemotherapy was stopped midway through the course and surgery was performed without the second course. Adverse events were evaluated according to NCI-CTCAE, version 3.0.

### Evaluating neoadjuvant chemotherapy response

Response to neoadjuvant chemotherapy was evaluated according to the Japanese Classification of Esophageal Cancer, 11th Edition [17].

Up to 5 large-sized lymph nodes were targeted from among 10-mm or more enlarged lymph nodes. Cases wherein the sum of the major diameters of the target lesions decreased by 30% or more were defined as PR, while those wherein the sum of the major diameters increased by 20% or more were defined as PD. The remaining cases were defined as SD.

Among cases who had no enlarged lymph nodes over 10 mm, response was determined based on the primary site of esophageal cancer. Cases wherein the major axis of the primary esophagus cancer decreased by 30% or more were defined as PR, while those wherein the major axis of the primary esophagus cancer increased by 20% or more were defined as PD. Moreover, cases wherein upper gastrointestinal endoscopy showed reduced or flattened tumor or ulcer margin ridges or thinned or clean ulcer bases were defined as PR, while others were defined as SD.

### Pathological response to chemotherapy

The degree of histopathological tumor regression in the surgical specimen was classified into four categories. The extent of viable residual carcinoma at the primary site was assessed semiquantitatively based on the estimated percentage of viable residual carcinoma in relation to the macroscopically identifiable tumor bed that was evaluated histopathologically. The percentage of viable residual tumor cells within the entire cancerous tissue was assessed as follows: grade 3, no viable residual tumor cells (pathological complete response); grade 2, less than one-third residual tumor cells; grade 1b, more than one-third but less than two-thirds residual tumor cells; grade 1a, more than 2/3 residual tumor cells; and grade 0, almost all residual tumor cells.

### Statistical analysis

The association between serum IgG levels and response to chemotherapy was assessed using the Mann–Whitney U test. The cutoff serum IgG levels were determined as the point maximizing the Youden index in the receiver operating characteristic (ROC) curve. Comparison between groups was performed using the chi-square test. A column with few cases was analyzed using Fisher’s exact test. Logistic regression analysis using the backward elimination technique to derive a potentially suitable set of predictors was performed for each outcome parameter. All statistical analyses were performed using EZR [18], a graphical interface for R, with *p*-values <0.05 indicating statistical significance. More precisely, EZR is a modified version of R commander designed to add statistical functions frequently used in biostatistics.

## Results

### Patient characteristics

Table 1 summarizes the clinical characteristics of cases included herein. Approximately 90% of the cases were male, with 31 (40%) receiving DCF. Moreover, 60 cases (71%) received two cycles of chemotherapy, whereas 25 (29%) received only one cycle due to severe adverse events.

**Table 1:** Clinical characteristics of patients and response to chemotherapy

Characteristics	
Age	68 (64,71)
Median (25%, 75% quartile)	
Sex, n	Aug-69
Male/Female	
Final T factor, n	6/18/14/36/3
T0/T1/T2/T3/T4	
Final N factor, n	34/14/24/4/1
N0/N1/N2/N3/N4	
Chemotherapy regimen, n	46/31
FP/DCF	
Cycles of chemotherapy, n	52/25
2/1	
Chemotherapy response, n	0/37/39/1
CR/PR/SD/PD	

### Response to neoadjuvant chemotherapy

Among the 77 patients included herein, 58 had swollen lymph nodes larger than 10 mm, with the response to chemotherapy being determined using the rate of change of the target lesions. In other patients, the response was determined using the primary esophageal lesion. Accordingly, 37 cases were classified as PR, 39 as SD, and 1 as PD, respectively. None of the cases were classified as CR. This study divided cases into two groups: the effective response group (PR) and ineffective response group (SD and PD).

### Chemotherapy regimen

The effective response group tended to have more cases receiving DCF, although no significant difference was noted (*p* = 0.067). However, the effective response group had significantly more cases who received two chemotherapy cycles than one cycle (*p* < 0.001).

### Relationship between chemotherapy response and serum IgG level

**Fig. 2:**
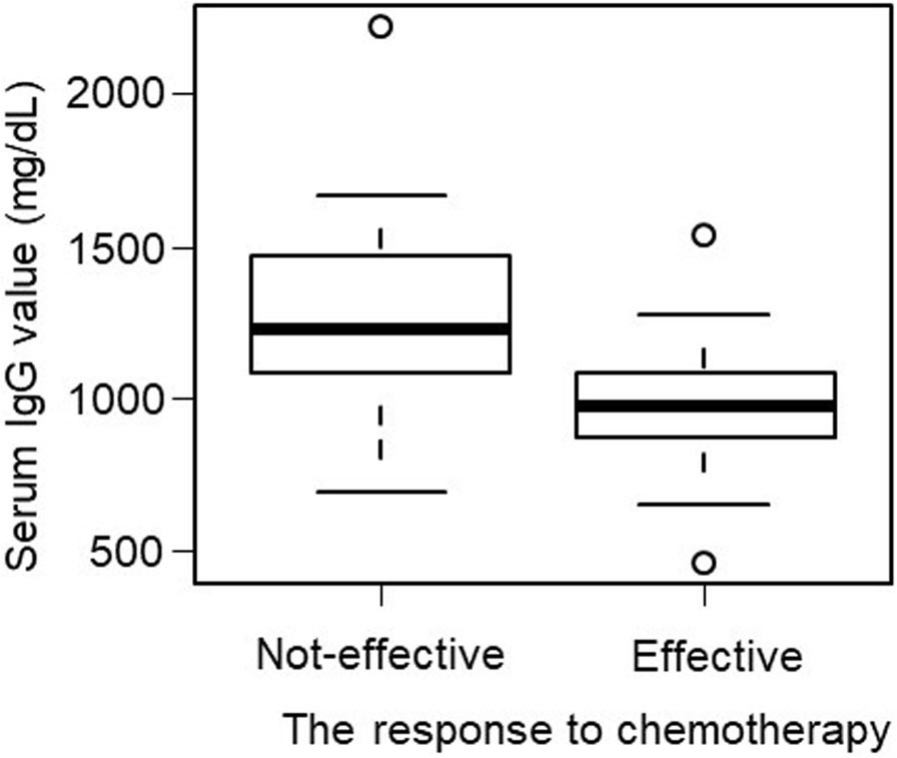
Association between serum IgG levels and response to chemotherapy

Patients included herein had a serum IgG level ranging from 460 to 2223 mg/dL. The median (25th percentile [Q1], 75th percentile [Q3]) serum IgG level in the effective (*n* = 37) and ineffective (*n* = 40) response groups were 976 (871, 1087) and 1227.5 (1087, 1466), respectively (Fig. 2). The effective group had a significantly lower serum IgG level than the ineffective group (*p* < 0.001).

**Fig. 3:**
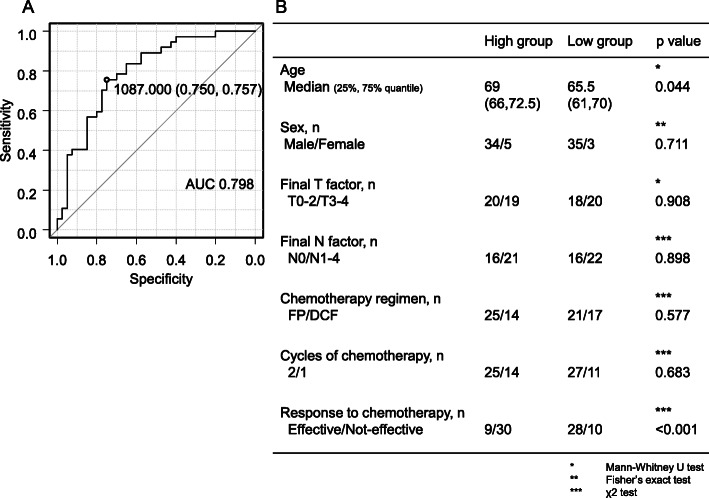
**A** Receiver operating characteristic (ROC) curve of serum IgG levels for predicting the response to chemotherapy. **B** Association between clinical characteristics and response to chemotherapy according to serum IgG levels

The ROC curve was created using “effective” as the target, with the area under curve being 79.8%. The cutoff serum IgG value at which the Youden index was maximized was determined to be 1087 mg/dL (sensitivity 0.750, specificity 0.757) (Fig. 3A).

Using the determined cutoff value, cases were then divided into high (39 cases) and low (38 cases) IgG groups. Although the high IgG group was older than the low IgG group, no significant differences in gender, final T factor, final N factor, chemotherapy regimen, and number of chemotherapy cycles were observed between both groups. The low IgG group had significantly more cases who had effective response to chemotherapy compared to the high IgG group (odds ratio [OR] = 9.009; 95% confidence interval [CI] = 2.974–30.157; *p* < 0.001) (Fig. 3B).

### Univariate and multivariate analyses

To identify independent predictors for the response to chemotherapy, clinicopathological factors were assessed using univariate and multivariate logistic regression analyses. Accordingly, univariate analysis identified age (OR = 0.237; 95% CI = 0.086–0.649; *p* = 0.005), neoadjuvant chemotherapy cycles (OR = 9.120; 95% CI = 2.720–30.600; *p* < 0.001), neutrophil count (OR = 0.210; 95% CI = 0.067–0.658; *p* = 0.007), lymphocyte count (OR = 0.095; 95% CI = 0.159–0.454; p = 0.003), and IgG levels (OR = 0.107; 95% CI = 0.038–0.302; *p* < 0.001) as significant predictors for the response to chemotherapy. Multivariate analysis was performed for the factors that showed significant differences in univariate analysis. Multivariate analysis showed that only neoadjuvant chemotherapy cycles (OR = 16.700; 95% CI = 2.990–93.300; *p* = 0.001) and IgG levels (OR = 0.085; 95% CI = 0.019–0.382; *p* = 0.001) were independent predictors for the response to chemotherapy in ESCC (Table 2).

**Table 2:** Univariate and multivariate analyses for factors predicting response to chemotherapy

		Univariate analysis			Multivariate analysis		
Factor	Unfavorable/favorable	Odds ratio	95% CI	*p* value	Odds ratio	95% CI	*p* value
Age	>65/65≥	0.237	0.086–0.649	0.005	0.517	0.128–2.090	0.354
NAC regimen	FP/DSF	0.406	0.160–1.030	0.059			
NAC cycles	1/2	9.12	2.720–30.600	<0.001	16.7	2.990–93.300	<0.001
Final T factor	T3-4/T0-2	1.6	0.652–3.950	0.304			
Final N factor	N1-4/N0	0.568	0.229–1.410	0.223			
Neutrophil	>3000/3000≥	0.21	0.067–0.658	0.007	0.298	0.071–1.240	0.097
Lymphocyte	>1800/1800≥	0.095	0.020–0.454	0.003	0.249	0.038–1.650	0.15
NLR	>2.645/2.645≥	0.4	0.159–1.010	0.052			
CRP	>0.26/0.26≥	0.393	0.148–1.040	0.06			
IL-6	>8.8/8.8≥	0.203	0.406–1.010	0.052			
IgG	>1087/1087≥	0.107	0.038–0.302	<0.001	0.085	0.019–0.382	<0.001

### Pathological findings

**Fig. 4:**
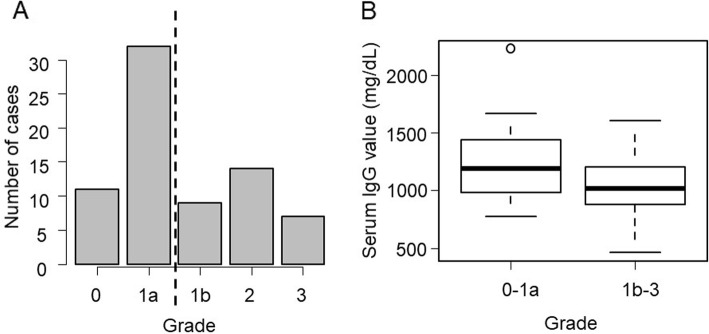
**A** Distribution of grades according to pathological findings. **B** Association between serum IgG levels and response to chemotherapy according to pathological findings

All cases underwent esophagectomy, after which the relationship between histopathological response and serum IgG value was investigated. The number of cases according to each grade is presented in Fig. 4A. Effective pathological response was defined as grade 1b or higher (less than two-thirds residual tumor cells). Accordingly, cases with effective pathological response had significantly lower pretreatment serum IgG levels compared to those who did not (*p* = 0.006) (Fig. 4B).

### Adverse events

Hematological toxicities are summarized in Table 3. Grade 3/4 neutropenia and lymphopenia were observed in 33 (43%) and 10 (13%) cases, respectively.

**Table 3:** Association between NAC regimens/cycles and adverse events (neutropenia and lymphopenia)

	Neutropenia		Lymphopenia	
	Grade 3	Grade 4	Grade 3	Grade 4
NAC regimen, n (%)				
FP	5 (11%)	1 (2%)	3 (7%)	0
DCF	11 (35%)	16 (52%)	7 (23%)	0
NAC cycles, n (%)				
1	1 (4%)	4 (16%)	5 (20%)	0
2	15 (27%)	13 (24%)	5 (9%)	0

The risk of grade 3/4 neutropenia was significantly higher in DCF than in FP (OR = 41.31; CI = 10.08–224.34; *p* < 0.001). Although not significantly different, the risk of grade 3/4 lymphopenia tended to be higher in DCF than in FP (OR = 4.10; CI = 0.84–26.84; *p* = 0.080). The risk of grade 3/4 neutropenia was significantly higher in 2 cycles of neoadjuvant chemotherapy than in 1 cycle (OR = 4.57; CI = 1.39–18.05; *p* = 007) (Table 3).

Serum IgG hypoplasia is not defined in NCI-CTCAE. The reference interval of serum IgG in adults is reported to be 665–2067 mg/dL [19]. A decrease in serum IgG level below the reference interval was observed in 10 cases (13%). The risk of decreased serum IgG levels was not significantly different in NAC regimen or the number of NAC (Table 4).

**Table 4:** Association between NAC regimens/cycles and serum IgG hypoplasia

	IgG < 665
NAC regimen, n (%)	
FP	4 (9%)
DCF	6 (19%)
NAC cycles, n (%)	
1	3 (13%)
2	7 (13%)

The medians of decrease rate after 2 weeks of NAC in neutrophil, lymphocyte, and IgG levels were 72.4%, 30.0%, and 16.7%, respectively.

## Discussion

While studies have shown that neoadjuvant chemotherapy is beneficial for improving the prognosis of stage II/III esophageal cancer [5], not all cases exhibit an effective response to chemotherapy. As such, predicting response before treatment may further improve the prognosis for esophageal cancer. Recently, a number of studies have attempted to predict the response to chemotherapy [11, 12, 15, 20], with their findings suggesting the utility of certain immunological factors.

Among the immunological factors, the current study focused on IgG levels. While reports have found that serum IgG was associated with prognosis in other carcinomas [21, 22], no study has shown a relationship between serum IgG levels and response to chemotherapy. Considering that serum IgG levels can be easily measured using blood tests prior to chemotherapy, it can therefore be an important factor for determining the subsequent treatment provided that it can predict the response to chemotherapy.

Previous reports have suggested that the NLR can be useful for predicting the response to chemotherapy in various malignant tumors [11, 12, 16]. The current study also found that immunological factors, such as neutrophil count, were useful for predicting response to chemotherapy, although IgG levels were more predictive and had been identified as an independent predictor.

Although the association between other immunological factors and response to chemotherapy has been established, it remains unclear why serum IgG levels are associated with response to chemotherapy. Given that immune function also depends on systemic inflammation status, patients with high immune disease may have suppressed anti-tumor activity of cytotoxic T lymphocytes, thereby promoting unsatisfactory response to chemotherapy. Reports have shown that high serum neutrophil levels were associated with systemic inflammation, not only immunity to cancer. Therefore, response to chemotherapy in such cases may be poor [11].

The current study found that chemotherapy was often ineffective in cases with high serum IgG levels, which is related not only to cancer immunity but also to systemic inflammation. As such, immunity to cancer may have been compromised in cases with systemic inflammation, resulting in poor response to chemotherapy.

One example of IgG-mediated immunity is ADCC, wherein immunoglobulin binds to the surface antigen of the tumor, and NK cells recognize its Fc region and binds to it, subsequently damaging tumor cells [23]. Regarding the association between ADCC activity and chemotherapy, trastuzumab for breast cancer has been shown to activate ADCC and exert anticancer effects [24]. Moreover, studies have shown that chemotherapy is effective in cases where lymphocyte tumor infiltration is high [25, 26].

In this study, there were more hematological toxicities in DCF than in FP. In addition, repeated NAC increased neutropenia. Compared with neutrophil and lymphocyte, serum IgG level was found to be less susceptible to chemotherapy. In univariate analysis, neutrophil and lymphocyte counts before chemotherapy were predictors of response to chemotherapy. However, in multivariate analysis, they were not independent predictors. Neutrophil and lymphocyte counts may be confounded with NAC regimen and cycles. In this study, when serious myelosuppression was observed, the dose of the second course was reduced or the second course of chemotherapy was not performed. Under the influence of age and underlying disease, neutrophils and lymphocytes contributed in the acceptance of chemotherapy. Serum IgG levels were an independent predictor, as they were less affected by the adverse events of chemotherapy.

## Conclusions

The current study found that serum IgG levels can predict the response to neoadjuvant chemotherapy among patients with esophageal squamous cell carcinoma. Considering that serum IgG value can be measured easily through blood tests. The current study has not revealed the mechanism. However, it is possible that IgG is involved in cancer immunity and chemotherapy. It may be an important factor in determining future treatment options for esophageal squamous cell carcinoma.

## Data Availability

The datasets used during the current study are available from the corresponding author on reasonable request.

## Abbreviations

ADCC: Antibody-dependent cellular cytotoxicity
DCF: Docetaxel, cisplatin, and 5-FU
ESCC: Esophageal squamous cell cancer
FP: 5-Fluorouracil and cisplatin
NLR: Neutrophil–lymphocyte ratio
